# “A star is born”—A unique case of an intranasal foreign body

**DOI:** 10.1007/s00508-023-02320-2

**Published:** 2024-01-04

**Authors:** Alexandros Andrianakis, Peter Valentin Tomazic

**Affiliations:** Department of Otorhinolaryngology, Head and Neck Surgery, Medical University of Graz, Graz, Austria

**Fig. 1:**
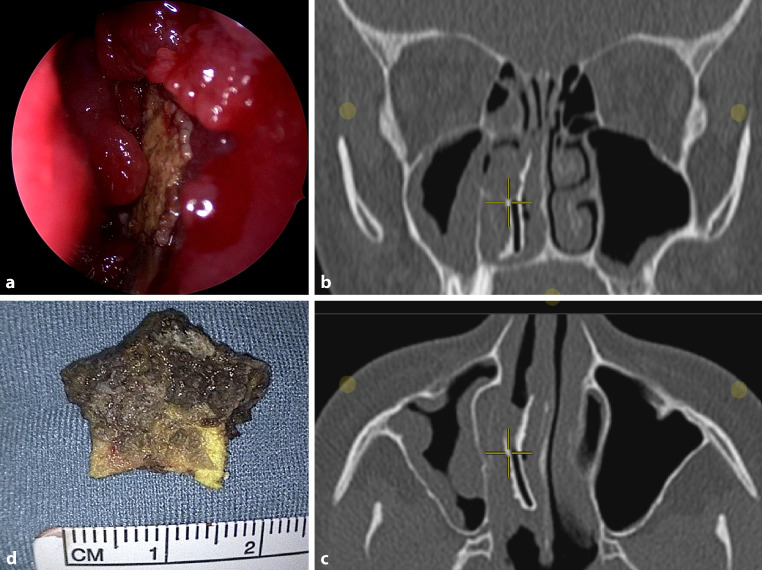
**a** Anterior rhinoscopy. **b**, **c** Computed tomography imaging of the paranasal sinuses (**b** coronal plane, **c** axial plane). The crosswire indicates a vertically situated hyperdense foreign body with surrounding soft tissue density in the right common middle meatus. **d** Removed foam star from the nasal cavity

A 16-year-old girl presented to the otorhinolaryngology outpatient clinic with a 10-year history of unilateral nasal airway obstruction and foul-smelling nasal discharge. The anterior rhinoscopy showed a large irregular mass in the nasal cavity consisting of extensive crusting and inflammatory granulation tissue (Fig. 1a). Computed tomography of the paranasal sinuses revealed a vertically situated hyperdense foreign body with surrounding soft tissue density in the right common middle meatus (Fig. 1b,c). Endoscopic sinus surgery with the patient under general anesthesia was further scheduled in order to remove the foreign body. During surgery, a yellowish-black foreign body in the shape of a star was successfully removed in toto (Fig. 1d). The respective foreign body was a foam star that has triggered a local inflammatory reaction and had developed into a rhinolith after some time. The star was probably put in the nose by the patient herself many years ago as a child, which she could not remember. The further postoperative follow-up remained free of complications and the patient no longer reports any sinus symptoms.

